# A Case of Phosphoglyceride Crystal Deposition Disease in the Pelvic Soft Tissues Recurring after Initial Surgery

**DOI:** 10.1155/2015/751582

**Published:** 2015-02-11

**Authors:** Yuki Yamada, Kazuhiro Nishioka, Hirotaka Kajihara, Taketoshi Noguchi, Katsuhiko Naruse, Kiyoshige Horie

**Affiliations:** ^1^Department of Obstetrics and Gynecology, Yamato-Takada Municipal Hospital, Yamato-Takada, Nara 6358501, Japan; ^2^Department of Obstetrics and Gynecology, Nara Medical University, Kashihara, Nara 6348521, Japan

## Abstract

Phosphoglyceride crystal deposition disease (PGDD) is a rare disease entity that is characterized by phosphoglyceride crystal deposition that stimulates the formation of masses in soft tissue scars or bones. We report a case of PGDD in the pelvic soft tissues that recurred after initial surgical treatment. A 50-year-old woman was referred to our hospital for the evaluation of pelvic masses that were observed on an abdominal ultrasound. Magnetic resonance imaging (MRI) revealed masses in the pelvic region, with the largest being 10 cm in diameter. The masses were diagnosed as ovarian malignant tumors, and an exploratory laparotomy was performed. Operative findings revealed them to be foreign body granulomas, and the patient was diagnosed with PGDD. The patient had a history of cesarean delivery at the age of 24 years. PGDD is extremely rare, but it should be considered in the differential diagnosis of abdominal masses in patients with a history of abdominal surgery.

## 1. Introduction

Phosphoglyceride crystal deposition disease (PGDD) is characterized by phosphoglyceride crystal deposition that stimulates neoplasia in soft tissue scars or bones [1]. As this condition is extremely rare, PGDD is sometimes strongly suspected of being a malignant tumor on the basis of clinical and radiographic findings. The distinguishing features of PGDD are slow growth, multiple spreads, and frequent recurrence after surgical resection [1, 2].

In this report, we describe a case of PGDD in the pelvic soft tissue, which was treated surgically after being initially misdiagnosed as ovarian malignant tumors and recurred twice.

## 2. Case Presentation

A 50-year-old Japanese woman was referred to our hospital for the evaluation of pelvic masses. This abnormality was observed on an abdominal ultrasound which she underwent as part of a routine check-up. The patient had a history of a cesarean delivery at the age of 24 years without any postoperative complications and had no significant family history.

**Figure 1:**
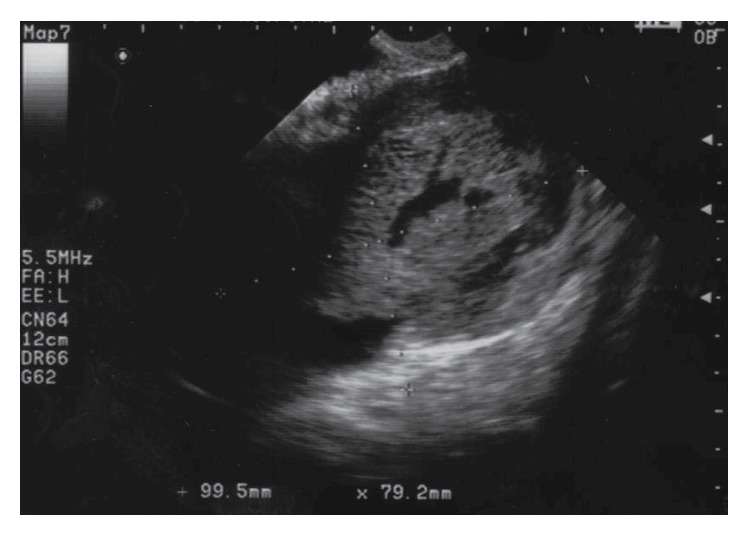
Transabdominal ultrasonographic image of the tumor. The mass was 10 cm in diameter, with a high echoic area in the center surrounded by a low echoic area.

On the first medical examination, the pelvic masses were detected by transabdominal ultrasound, and the largest mass was approximately 10 cm in diameter (Figure 1). All laboratory parameters, including major tumor markers (carcinoembryonic antigen (CEA), carbohydrate antigen (CA) 125, CA19-9, squamous cell carcinoma antigen (SCC), and alpha-fetoprotein (AFP)), were within normal limits.

**Figure 2:**
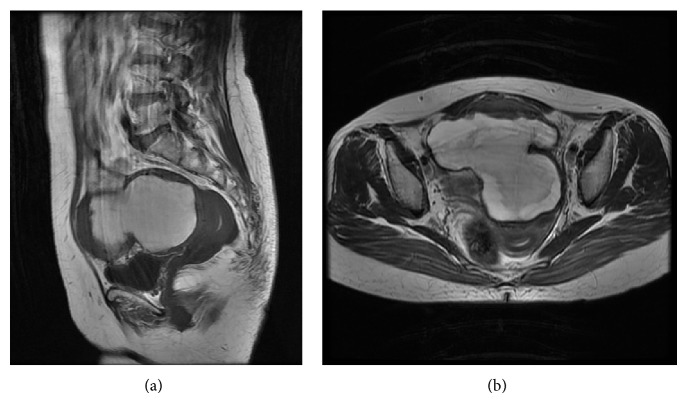
Magnetic resonance imaging of the pelvis. Lobulated masses in the pelvic region (the largest being 10 cm in diameter) that contained a hemorrhagic component and a solid part at the center that exhibited a low intensity on T2-weighted images are shown in both (a) sagittal (T1WI) and (b) transverse (T2WI) sections.

Magnetic resonance imaging (MRI) also revealed lobulated masses in the pelvic region, with the largest being 10 cm in diameter. The masses contained a hemorrhagic component, as well as a central solid part that exhibited a low intensity on T2-weighted images (Figures 2(a) and 2(b)). Some small masses were present at the Douglas cavity, and these were suspected to be disseminations of the malignant tumor. Since both ovaries were not detected, an exploratory laparotomy was performed under the diagnosis of ovarian malignant tumors.

Masses that adhered to the parietal peritoneum and the bladder were detected at the vesicouterine pouch. A part of the omentum had also adhered to the masses, and some swollen omental lymph nodes were observed. Dissemination-like small masses, which had torn from the omentum and the peritoneum, were also detected at the peritoneum of the vesicouterine pouch. Other masses were detected at the Douglas cavity, but these were strongly attached to the rectum, making excision impossible. Both ovaries were normal sized, but a small dissemination was detected on the surface of the right ovary. A tumor of peritoneal origin was suspected, and tumor debulking, bilateral salpingooophorectomy, and omentectomy were performed.

**Figure 3:**
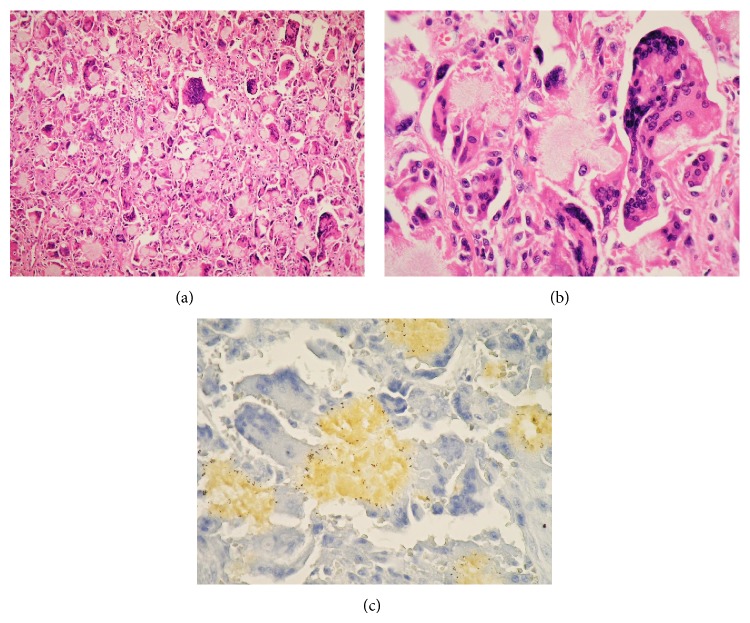
Histological findings. ((a) and (b)) Hematoxylin and eosin stains showing a foreign body granuloma consisting of string-like crystals and a foreign body giant cell, at the center of which was necrotic tissue with fibrin-like tissue and a cholesterol cleft. (c) Gold hydroxamic acid staining (reaction) for the detection of phosphoglyceride. String-like crystals are positively stained. Original magnification: (a) 100x and ((b) and (c)) 400x.

A pathologic examination revealed that these masses were foreign body granulomas consisting of string-like crystals and a foreign body giant cell, the center of which contained necrotic tissue with fibrin-like tissue and a cholesterol cleft (Figures 3(a) and 3(b)). On immunohistochemistry, the granuloma stained positive for CEA, CA125, cytokeratin, *α*1-antichymotrypsin, and MIB-1 (10%) but negative for p53 antibody. These masses were diagnosed as granulomas, without a mixture of epithelial cells or other cell components. The central crystal was determined to be phosphoglyceride by the gold hydroxamic acid method (Figure 3(c)).

This patient underwent regular follow-up abdominal ultrasound examinations after the first operation. A recurrence near the peritoneum was confirmed 4 years after the first operation, and the mass had grown to 5 cm in diameter, necessitating repeated surgery. Silk suture strings were used in the previous procedure, but as these were a possible cause of the recurrence, absorbable strings were used in the second operation. Four years later (8 years after the initial operation), a mass with a diameter of 3 cm was detected at the vesicouterine pouch and was considered to be the same granuloma. The mass was observed on ultrasound in an outpatient clinic, but irritative bladder symptoms had appeared with its growth, and an MRI revealed a mass 5 cm in diameter at the vesicouterine pouch. The patient's symptoms worsened during the observation period, and she was operated on the third time. Since the masses strongly adhered to the uterus, a total hysterectomy was performed. An ileal stricture was detected, and an ileectomy was also performed. All excised masses were diagnosed as PGDD. At 18 months since her last operation, the patient has had no symptoms of recurrence.

## 3. Discussion

PGDD is a rare disease defined as phosphoglyceride crystals deposited as tumors in the soft tissue or bone with no relation to the joint [1]. Phosphoglycerides are the major lipid component of all membranes. Macrophages at the deposition sites may take up phosphoglycerides and slowly degrade them. Some phosphoglycerides are insoluble in aqueous media, suggesting that some undegraded portions are kept in the lysosomes and contribute to the formation of the crystals' nidus. The nidus may gradually grow in size by upregulating similar phosphoglycerides to form visible crystals. However, the initial step of nidus formation occurs rarely in normal people, and even if it does occur, it may be easily cleared. One proposed hypothesis postulates that a localized disturbance of phosphoglyceride metabolism within the macrophages may be initiated by local inflammation, leading to a progressive amplification of macrophage infiltration and crystal deposition [2]. This hypothesis, however, has yet to be substantiated.

Histologically, PGDD is a foreign body granuloma consisting of crystals. Many histiocytes and macrophages surround the crystal and form foreign body granulomas. The crystals are 50–150 *μ*m in diameter, appearing as oval pink or blue aggregates on hematoxylin and eosin staining, and are arranged in corona-like circles. On polarized light microscopy, the fibrillar crystals appear refractive. The crystals do not dissolve in the usual specimen creation process, characteristically dissolve in acetic acid with oxygen gas formation, easily dissolve in alkalis, and stain positive for phosphoglycerides by the gold hydroxamic acid method.

There are other deposition diseases such as gout, pseudogout, or calcium pyrophosphate. Although most of the above crystals accumulate in and around joints, phosphoglyceride crystals have shown no association with the joints and exhibit characteristics quite different from those of the above. A large mass can sometimes form and be misdiagnosed as a malignant tumor. To our knowledge, only 8 cases of this deposition disease have been reported previously, and little is known of the etiology and pathogenesis of this disorder. No sex predilection, congenital abnormalities, or family history of metabolic disorders has been noted in connection with this disease entity. Deposition sites were characteristically intramuscular injection sites or postoperative sites including the buttock muscle [3], brachial muscle, spine [4], and abdominal soft tissue [5]. The masses gradually increased in size over more than 10 years in the soft tissue at the postoperative locus and were detected by routine check-up or subsequent to the appearance of symptoms. Therefore, patients have generally been older than 50 years.

In this case, an exploratory laparotomy was performed for a suspected malignant tumor of the ovary by MRI. This patient had a history of cesarean section at the age of 24 years, and local inflammation at the operation site was suspected to have led to a progressive amplification of macrophage infiltration and crystal deposition. The previous cases in former reports [2, 3] were also diagnosed as neoplasms, and excisional surgery was performed due to the difficulty of radiographically discriminating between benign masses and malignancies. In this case, an exploratory laparotomy was first performed, and an attempt was made to observe the recurrent masses 4 years later, because the next operation could be a causative factor for new lesions. However, the patient's symptoms worsened during observation and debulking surgery was needed. Recurrence was confirmed twice after the initial surgery, and additional surgeries were performed twice to remove the deposited masses. Thus, we emphasize that patients with PGDD require careful follow-up.

PGDD is an extremely rare condition, but it should be considered in the differential diagnosis of abdominal masses in patients with a history of abdominal surgery.
